# Transcriptomic Profiling during the Post-Harvest of Heat-Treated Dixiland *Prunus persica* Fruits: Common and Distinct Response to Heat and Cold

**DOI:** 10.1371/journal.pone.0051052

**Published:** 2012-12-06

**Authors:** Martin A. Lauxmann, Bianca Brun, Julia Borsani, Claudia A. Bustamante, Claudio O. Budde, María V. Lara, María F. Drincovich

**Affiliations:** 1 Centro de Estudios Fotosintéticos y Bioquímicos (CEFOBI), Universidad Nacional de Rosario, Rosario, Argentina; 2 Estación Experimental San Pedro, Instituto Nacional de Tecnología Agropecuaria (INTA), San Pedro, Argentina; University of New England, Australia

## Abstract

Cold storage is extensively used to slow the rapid deterioration of peach (*Prunus persica* L. Batsch) fruit after harvest. However, peach fruit subjected to long periods of cold storage develop chilling injury (CI) symptoms. Post-harvest heat treatment (HT) of peach fruit prior to cold storage is effective in reducing some CI symptoms, maintaining fruit quality, preventing softening and controlling post-harvest diseases. To identify the molecular changes induced by HT, which may be associated to CI protection, the differential transcriptome of peach fruit subjected to HT was characterized by the differential display technique. A total of 127 differentially expressed unigenes (DEUs), with a presence-absence pattern, were identified comparing peach fruit ripening at 20°C with those exposed to a 39°C-HT for 3 days. The 127 DEUs were divided into four expression profile clusters, among which the heat-induced (47%) and heat-repressed (36%) groups resulted the most represented, including genes with unknown function, or involved in protein modification, transcription or RNA metabolism. Considering the CI-protection induced by HT, 23-heat-responsive genes were selected and analyzed during and after short-term cold storage of peach fruit. More than 90% of the genes selected resulted modified by cold, from which nearly 60% followed the same and nearly 40% opposite response to heat and cold. Moreover, by using available *Arabidopsis* microarray data, it was found that nearly 70% of the peach-heat responsive genes also respond to cold in *Arabidopsis*, either following the same trend or showing an opposite response. Overall, the high number of common responsive genes to heat and cold identified in the present work indicates that HT of peach fruit after harvest induces a cold response involving complex cellular processes; identifying genes that are involved in the better preparation of peach fruit for cold-storage and unraveling the basis for the CI protection induced by HT.

## Introduction

Peach (*Prunus persica* L. Batsch) is one of the most popular fruit in the world due to its high nutritional value and pleasant flavor. After harvest, cold storage is extensively used to slow the rapid deterioration of this fruit at ambient temperature since it reduces enzymatic activity and slows down the respiratory rhythm, allowing the shipping and marketing. Nonetheless, peach fruits that are subjected to long periods of cold storage develop chilling injury (CI) symptoms [1]. Internal and external browning, lack of juiciness (mealiness or woolliness), flesh break-down, black pit cavity, loss of flavor and inability to ripen, are some of the CI symptoms. Remarkably, CI disorders usually become visible when fruits reach the consumers, turning fruits unpalatable and leading to consumer rejection.

Several studies have investigated the biochemical basis of CI disorders in peach by analyzing transcriptomic changes under cold storage. For this, macroarray [2] and microarray technology [3], [4] and EST transcript profiling [5] of peach fruit under different cold storage conditions allowed the identification of cold-responsive genes that may be involved in the development of some CI disorders. In addition, previous studies on cell wall-modifying enzymes under cold storage of peach revealed the importance of pectin metabolism in the generation of CI disorders [6]. Besides, proteomic analysis of peach fruit under CI-induced storage was also performed. By this technique, thaumantin-like proteins [7] and proteins involved in stress response [8] were postulated as playing roles in the protection against CI. Moreover, proteins involved in membrane stability were identified [9], reinforcing the role of lipid unsaturation in the acquisition of chilling tolerance in peach fruit [10], [11].

In order to alleviate CI symptoms and extent fruit post-harvest life, a wide variety of different pre- or post-harvest treatments have been applied to peach. Heat treatments (HT), controlled atmosphere storage, or application of chemical compounds (as salicylic acid, methyl jasmonate, γ-aminobutyric acid or gibberellic acid) have been successfully used alone or in combination when applied prior to cold-storage [12]–[20]. Among the wide variety of treatments, HT is one of the most extensively studied procedure [21]–[29]. Moreover, HT represents a feasible treatment for commercial application, being also effective for managing post-harvest decay [30]. Other relevant feature of HT is that, although it delays the ripening process, ripening is recovered upon return to moderate temperature, with minor changes in biochemical processes [14], [31].

One way to address the molecular mechanisms associated to CI protection is the analysis of the molecular changes associated to a treatment that avoids or decreases CI symptoms. In this regard, it has been shown that the alleviation of some of the CI symptoms by the application of some chemical compounds is linked to the induction of antioxidant systems or Heat Shock proteins (HSPs) [12], [17]–[19]. With regards to HT, proteomic studies have identified induced or repressed proteins by this treatment, which may be involved in CI protection when this treatment precedes the following chilling [14], [32]. However, the transcriptomic changes induced by HT that leads to the protecting mechanisms against CI have not been analyzed yet and may be useful in the understanding of the molecular networks induced by HT. Thus, in the present study, the differential transcriptome of Dixiland peaches subjected to a three-day-HT at 39°C after harvest [14] was characterized by the differential display (DD) technique. More than one hundred genes with differential pattern of expression, which are involved in diverse biological processes, were identified. These genes were further analyzed regarding their expression in cold-treated peach fruit and *Arabidopsis*, identifying several genes that converge in the response to heat and cold signals and that are thus involved in the CI protection induced by HT. The biological implications of HT post-harvest technology applied to CI protective mechanism are discussed for specific identified genes.

## Results

### Differential Transcript Level after Heat Treatment of Dixiland Peach Fruit

**Figure 1:**
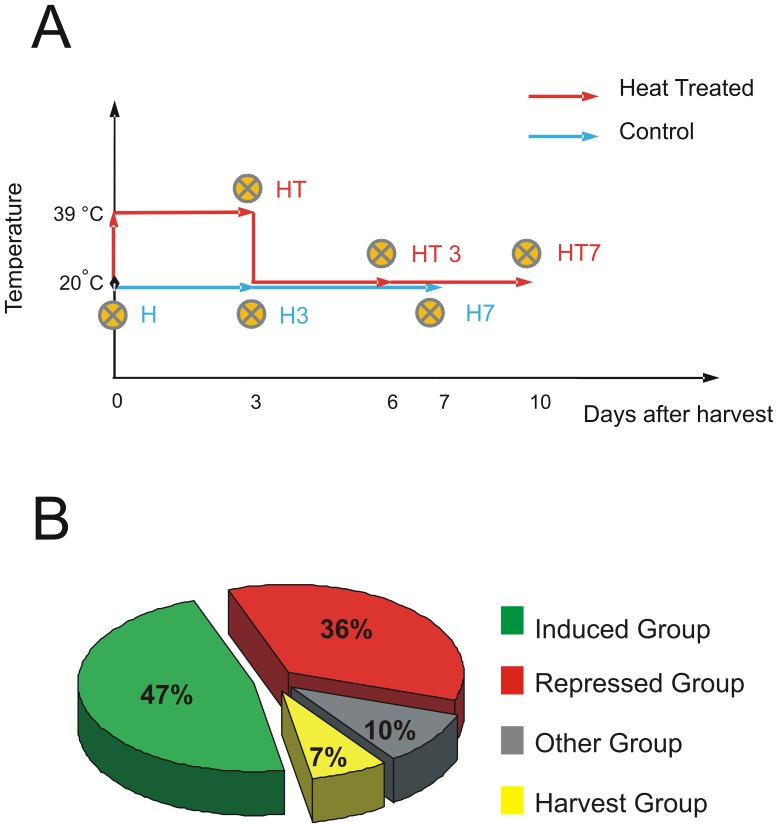
Treatments of peach fruits and clustering of the differential transcriptome. A. Schematic representation of Dixiland peach fruit samples employed for the transcriptomic analysis. The differential transcriptome was obtained comparing fruits at six different stages: harvest time (H); after 3 and 7 days of harvest (H3 and H7, respectively) along the normal ripening process (light blue arrows) at 20°C; after the application of a heat treatment (upper red arrow) of 39°C during 3 days (HT) and after 3 and 7 days (HT3 and HT7, respectively) at 20°C after the heat treatment (lower red arrows) at 20°C. Both H3 and HT fruits have the same post-harvest lifetime. H7 and HT3 fruits have approximately the same post-harvest lifetime. However, HT7 fruits have no control counterparts of the same post-harvest lifetime. Crossed circles indicate the time-points of sample collection for the analysis. B. Pie chart representing the four clusters of unigenes of the differential transcriptome. Induced- and repressed-upon-heat-treatment unigenes were classified according to their expressional pattern (Table 1) and gathered into the IG and RG, respectively. These clusters represent 83% of the differential post-harvest transcriptome. OG includes unigenes whose differential expression pattern can not be related to the applied treatment. HG includes unigenes expressed at harvest stage and that are repressed during the ripening of fruits.

In the present work, the differential transcriptome of Dixiland peach fruit was analyzed by comparing six different samples (Figure 1A): harvest time (H); after 3 and 7 days along the normal ripening process at 20°C (H3 and H7, respectively); after the application of a heat treatment of 39°C for 3 days (HT); and after 3 and 7 days at 20°C after the heat treatment (HT3 and HT7, respectively). The principal quality parameters of the different peach samples are indicated in Table S1. A total of 145 differential expressed transcripts (DETs), ranging in size from approximately 100 to 800 bp and showing a presence-absence pattern, were successfully isolated and sequenced. Over-represented DETs were organized as contigs to eliminate redundancy giving a total of 127 unigenes that are differential expressed (DEUs) among the analyzed samples.

**Table 1:** Clustering of the identified differentially expressed transcripts (DETs) and summary of the total differentially expressed unigenes (DEUs).

Cluster identity	Total Unigenes	Type of expression pattern	Number of DETs	Number of DEUs
**Induced group (IG)**	**60**	000100	58	50
		001111	4	4
		000111	3	3
		000011	2	2
		001100	1	1
**Repressed group (RG)**	**45**	111011	51	42
		111000	1	1
		011000	1	1
		001000	1	1
**Other patterns group (OG)**	**13**	000001	4	4
		100100	4	4
		001010	2	2
		011111	2	2
		010010	1	1
**Harvest group (HG)**	**9**	100000	10	9

The type of expression pattern consists of a six-number sequence which represents the absence (0) or presence (1) of a transcript in the control ripening conditions (H, H3 and H7, respectively) and after the application of HT and along the ripening after the treatment (HT, HT3 and HT7, respectively).

### Clustering Analysis of the Differential Expressed Unigenes

The expression patterns of the DEUs identified were binary typified, assigning the number one (1) to the presence of the transcript in a particular post-harvest condition and zero (0) to the absence of it (Table 1). Thus, each binary-coded pattern consisted of a six-number sequence; the first three numbers represent the absence (0) or presence (1) at harvest and during the control ripening conditions (H, H3 and H7, respectively) and the last three ones indicate the absence (0) or presence (1) after the application of HT and along the ripening after the treatment (HT, HT3 and HT7). By using this binary coded-pattern, 15 different expression patterns were detected among the 127 DEUs (Table 1).

**Table 2:** Functional identity and gene ontology description of the isolated unigenes corresponding to the “Induced Group” (IG) unigenes.

Unigene Clone GenBank #	NCBI accession no.	NCBI and ESTreeDB functional annotation	TAIR Unigene functional annotation	*Arabidopsis* ortholog id
**Expression pattern: 000100**				
**I1 JK845705**	XM_002281082	Transcription factor bHLH110-like (*Vitis vinifera*).	Basic helix-loop-helix (bHLH) DNA-binding superfamily protein	At5g09750
**I2 JK845706**	DQ848603	AUX/IAA27 A mRNA, partial cds (*Malus domestica*).	IAA27, PAP2 (Phytochrome-Associated Protein 2).	At4g29080
**I3 JK845707**	XM_002533657	Putative 40S ribosomal protein S7 (*Ricinus communis*).	40S ribosomal protein S7 (RPS7C)	At5g16130
**I4 JK845708**	HM362681	Pyruvate decarboxylase (PDC) (*Prunus dulcis x P. persica*).	Thiamine pyrophosphate dependent pyruvate decarboxylase family protein.	At4g33070
**I5 JK845709**	HM122443	ARF domain class transcription factor (IAA2) mRNA(*M. domestica*).	RNA binding protein.	At4g14550
**I6 JK845710**	CU227747	EST from severe drought-stressed leaves (*Populus*).	P-loop containing nucleoside triphosphate hydrolases superfamily protein.	At4g16680
**I7 JK845711**	FJ752239	Monodehydroascorbate reductase (*M. domestica*).	Putative monodehydroascorbate reductase, ATMDAR1.	At3g52880
**I8 JK845712**	AY347830	LYTB-like protein mRNA (*M. domestica*).	CLB6, ISPH, HDR (4-Hydroxy-3-methylbut-2-enyl Diphosphate Reductase).	At4g34350
**I9 JK845713**	XM_002310083	Predicted protein (*Populus trichocarpa*).	Ubiquitin-protein ligase SKIP2, VFB4 (SKP1/ASK1 Interacting Protein 2).	At5g67250
**I10 JK845714**	Z70240	Cytochrome oxidase subunit III mRNA (*Olea europaea*).	COX3 (Cytochrome c Oxidase subunit 3).	Atmg00730
**I11 JK845715**	XM_002509648	Glucose-methanol-choline (gmc) oxidoreductase (*R. communis*).	Glucose-methanol-choline (GMC) oxidoreductase family protein.	At5g51950
**I12 JK845716**	FQ385429	Clone SS0AEB32YE13, NADH dehydrogenase ubiquinone (*V. vinifera*).	FRO1 (NADH dehydrogenase ubiquinone).	At5g67590
**I13 JK845717**	XM_003537865	Uncharacterized protein LOC100500529 (*Glycine max*).	CSL zinc finger domain-containing protein.	At2g15910
**I14 JK845718**	NM_001159207	Ferredoxin (LOC100286320) (*Zea mays*).	2Fe-2S ferredoxin-like superfamily protein.	At3g07480
**I15 JK845719**	XM_003526896	Uncharacterized protein LOC100813123(*G. max*).	DEK domain-containing chromatin associated protein.	At5g63550
**I16 JK845720**	XM_002283678	Uncharacterized LOC100264568 (*V. vinifera*).	Protein of unknown function (DUF677).	At5g66660
**I17 JK845721**	XM_002880653	Hypothetical protein (*Arabidopsis lyrata)*.	Protein of unknown function (DUF581).	At2g25690
**I18 JK845722**	AJ243415	Phosphoenolpyruvate carboxylase partial mRNA (*P. persica*).	ATPPC3 (Phosphoenolpyruvate Carboxylase 3).	At3g14940
**I19 JK845723**	NM_112918	*A. thaliana* lipid-binding serum glycoprotein family protein (AT3G20270) mRNA	Lipid-binding serum glycoprotein family protein.	At3g20270
**I20 JK845724**	XM_002318999	Histidine phosphotransfer protein (*P. trichocarpa*).	Putative two-component phosphorelay mediator.	At4g04402
**I21 JK845725**	XM_003526187	60S ribosomal protein L39-2-like LOC100779071 (*G. max*).	60S ribosomal protein L39 (RPL39C).	At4g31985
**I22 JK845726**	XM_002269267	ATP-dependent helicase rhp16-like (*V. vinifera*).	Helicase protein with RING/U-box domain.	At1g05120
**I23 JK845727**	Z86091	TCTP protein mRNA (*Fragaria x ananassa*).	TCTP (Translationally Controlled tumor protein).	At3g16640
**I24 JK845728**	AF139497	Homeobox leucine zipper protein (HBLZP) mRNA(*P. armeniaca*).	ATHB-3 (*Arabidopsis thaliana* Homeobox 3).	At5g15150
**I25 JK845729**	HQ853493	MLO1 protein mRNA (*M. toringoides*).	MLO8 (Seven transmembrane MLO family protein).	At2g17480
**I26 JK845730**	NM_001253187	E3 ubiquitin-protein ligase MARCH1-like (*G. max*).	RING/FYVE/PHD zinc finger superfamily protein.	At1g50440
**I27 JK845731**	XM_003553739	UPF0667 protein C1orf55 homolog (*G. max*).	Ubiquitin-like superfamily protein.	At4g01000
**I28 JK845732**	AY727482	PsbZ (psbZ) gene, complete cds and tRNA-Gly (trnG) gene (*P. nigra*).	PsbZ gene.	Atcg00300
**I29 JK845733**	XM_002313303	Predicted protein (*P. trichocarpa*).	RNA-binding (RRM/RBD/RNP motifs) family protein.	At3g20890
**I30 JK845734**	XM_002301724	Predicted protein (*P. trichocarpa*).	Glycosyl hydrolase family 38 protein.	At5g66150
**I31 JK845735**	XM_002319842	Predicted protein (*P. trichocarpa*).	Chloroplast-encoded 23S ribosomal RNA.	Atcg01180
**I32 JK845736**	FN395072	Partial mRNA for 1,3-beta-glucan synthase (cals2 gene) (*M. domestica*).	CALS1 (callose synthase 1)	At1g05570
**I33 JK845737**	EU494969	Xyloglucan endotransglucosylase/hydrolase 10 mRNA (*Malus x domestica*).	EXGT-A3 (endoxyloglucan transferase A3).	At2g01850
**I34 JK845738**	Z21794	Ribulose-1,5-bisphosphate carboxylase/oxygenase activase mRNA (*M. domestica*).	RCA (RubisCo Activase).	At2g39730
**I35 JK845739**	AF298827	60S ribosomal protein L24 (RL24) mRNA (*Prunus avium*).	STV1, RPL24 (Ribosomal protein L24e family Protein).	At3g53020
**I36 JK845740**	EU586193	SKP1-like protein 1 mRNA, cultivar Fuji (*M. domestica*).	ASK2, SKP1B (E3 ubiquitin ligase SCF complex subunit).	At5g42190
**I37 JK845741**	XM_002303907	Predicted protein, mRNA (*P. trichocarpa*).	OLEO1 (Oleosin 1).	At4g25140
**I38 JK845742**	XM_002303301	Predicted protein, mRNA (*P. trichocarpa*).	O-fucosyltransferase family protein.	At5g50420
**I39 JK845743**	EU627002	Heat-shock protein 70 (HSP70) mRNA*(Hevea brasiliensis*)	HSP70 (heat shock protein 70).	At3g12580
**I40 JK845744**	XM_002516198	Polygalacturonase, putative, mRNA (*R. communis*).	Glycoside hydrolase family 28 protein/polygalacturonase (pectinase) family protein.	At1g19170
**I41 JK845745**	XM_002279748	Hypothetical protein LOC100262831 (*V. vinifera*).	PEX10 (peroxin 10).	At2g26350
**I42 JK845746**	AK324359	cDNA, clone: LEFL1076DE11, HTC in leaf (*Solanum lycopersicum*).	ZAT12 (C2H2-type zinc finger family protein).	At5g59820
**I43 JK845747**	XM_002276377	Vacuolar protein sorting-associated protein 2 homolog 3-like (V. *vinifera*).	VPS2.3 (Vacuolar Protein Sorting-associated protein 2.3).	At1g03950
**I44 JK845748**	AK326408	cDNA, clone LEFL2006J14 (*S. lycopersicum*).	WRKY40 (WRKY DNA-binding protein 40).	At1g80840
**I45 JK845749**	AF071892	Omega-6 fatty acid desaturase (O6FAD) mRNA (*P. armeniaca*).	FAD2 (Fatty Acid Desaturase 2).	At3g12120
**I46 JK845750**	XM_003552047	DEAD-box ATP-dependent RNA helicase 38-like (*G. max*).	LOS4 (P-loop containing nucleoside triphosphate hydrolases superfamily protein).	At3g53110
**I47 JK845751**	XM_002527140	Putative nucleotide binding protein mRNA (*R. communis*).	Transducin family protein/WD-40 repeat family protein.	At3g33530
**I48 JK845752**	XM_002531473	Putative histone h1/h5 (*R. communis*).	HIS1-3, Histone H1-3.	At2g18050
**I49 JK845753**	XM_002280711	Probable receptor-like protein kinase (*V. vinifera*).	SNC4 (suppressor of npr1-1 constitutive 4).	At1g66980
**I50 JK845754**	AF467900	Hypothetical protein clone pPn31C7, and hypothetical transcription factor (*P. persica*).	ATP-dependent RNA helicase.	At3g26560
**Expression pattern: 001111**				
**I51 JK845755**	CU223716	EST from leaves (*P. trichocarpa*).	ACBP6 (Acyl-CoA-Binding Protein 6).	At1g31812
**I52 JK845756**	XM_002523147	Nuclear transcription factor Y subunit A-7-like (*V. vinifera*).	NF-YA4 (Nuclear Factor Y, subunit A4).	At2g34720
**I53 JK845757**	HM122587	HD domain class transcription factor (HD8) mRNA (*M. domestica*).	Disease resistance protein (TIR-NBS-LRR class) family.	At5g51630
**I54 JK845758**	GU451710	14-3-3h protein mRNA (*Gossypium hirsutum*).	GRF9 (General Regulatory Factor 9), GF14 MU.	At2g42590
**Expression pattern: 000111**				
**I55 JK845759**	HM122540	DOF domain class transcription factor (DOF1) (*M. domestica*).	Dof-type zinc finger DNA-binding family protein.	At1g28310
**I56 JK845760**	XM_002519482	Putative carboxy-lyase (*R. communis*).	Putative lysine decarboxylase family protein.	At5g11950
**I57 JK845761**	AB289902	S gene for S-RNase, partial cds (*Prunus speciosa*).	Homeodomain-like superfamily protein.	At2g02060
**Expression pattern: 000011**				
**I58 JK845762**	AF220202	Soluble inorganic pyrophosphatase mRNA (*M. domestica*).	PPa4 (pyrophosphorylase 4).	At3g53620
**I59 JK845763**	XM_003532867	Uncharacterized protein LOC100795943 (*G. max*).	Protein of unknown function (DUF604).	At5g41460
**Expression pattern: 001100**				
**I60 JK845764**	DQ222997	Temperature-induced lipocalin (TIL) mRNA (*P. persica*).	TIL (Temperature-Induced Lipocalin).	At5g58070

The 127 DEUs were further divided into four different clusters (Table 1). The “Induced Group (IG)” represents the 47% of the total differential transcriptome (Figure 1B). It is composed of 60 unigenes that are induced by the applied HT, that is, they depict a presence or “up regulation” expressional pattern (000100, 001111, 000111, 000011 and 001100) after HT (Table 1). The second group, the “Repressed Group (RG)” represents the 36% of the total differential transcriptome (Figure 1B). This cluster is formed by 45 unigenes that are repressed upon the applied HT, having an absence or “down regulation” expressional pattern (111011, 00100, 011000 and 111000) (Table 1). Together, the induced and repressed clusters, account for the 83% (105 unigenes) of the differential post-harvest transcriptome. This important group of unigenes represents the “heat-responsive genes” (Tables 2 and 3). The third group, “Other-pattern Group (OG)” includes those unigenes that although they have differential transcriptional profiles, they are not induced or repressed by the applied treatment during the post-harvest lifetime. The OG represents 10% of the total differential transcriptome (Figure 1B) and includes 9 unigenes whose differential expressional profiles remain unchanged upon the HT in comparison with fruits of the same post-harvest age or unigenes that are delayed by the applied treatment (100100, 010010, 001010 and 011111) (Tables 1 and 4). The four unigenes with the expressional profile 000001 are also included into this cluster (Table 4). This pattern can not be exclusively associated to the HT itself since at HT7 lifetime peach fruits may undergo the senescence process instead of the ripening one(10 days after harvest, Figure 1A). The fourth cluster (Harvest Group “HG”) includes those differentially expressed unigenes that are expressed only at the harvest stage (expressional pattern 100000) representing only 7% of the total differential transcriptome (Figure 1B, Tables 1 and 5).

**Table 3:** Functional identity and gene ontology description of the isolated unigenes corresponding to the “Repressed Group” (RG) unigenes.

Unigene Clone GenBank #	NCBI accession no.	NCBI and ESTreeDB functional annotation	TAIR Unigene functional annotation	*Arabidopsis* ortholog id
**Expression pattern: 111011**				
**R1 JK845765**	XM_002275269	Lysine-specific demethylase REF6-like (*V. vinifera*).	REF6 (Relative of Early Flowering 6).	At3g48430
**R2 JK845766**	XM_002284516	calcium-transporting ATPase 4, endoplasmic reticulum-type-like (*V. vinifera*).	ECA4 (calcium-transporting ATPase).	At1g07670
**R3 JK845767**	AF179295	Putative actin depolymerizing factor (ADF) mRNA (*M. domestica*).	ADF4 (Actin Depolymerizing Factor 4).	At5g59890
**R4 JK845768**	EF534108	Chloroplast sequence (*Beta vulgaris*).	PCLPP (plastid-encoded CLP P).	Atcg00670
**R5 JK845769**	XM_002279489	Hypothetical protein LOC100246828 (*V. vinifera*).	PLAC8 family protein.	At4g23470
**R6 JK845770**	XM_002318247	Predicted protein (*P. trichocarpa*).	PME1 (Pectin Methylesterase Inhibitor 1).	At4g12390
**R7 JK845771**	AF086759	Uncharacterized protein LOC100801770 (*G. max*).	ESP1 (hydroxyproline-rich glycoprotein family protein).	At1g73840
**R8 JK845772**	XM_002321350	Predicted protein (*P. trichocarpa*).	Yippee family putative zinc-binding protein.	At4g27740
**R9 JK845773**	BT051272	Clone MTYF1_F2_F3_F41G-D-3 unknown mRNA (*Medicago truncatula*).	J8 (Chaperone DnaJ-domain superfamily protein).	At1g80920
**R10 JK845774**	DQ34532	Eukaryotic translation initiation factor eIF5A (*Rosa chinensis*).	eIF-5A 1 (Eukaryotic translation initiation factor 5A-1).	At1g26630
**R11 JK845775**	XM_003549582	Serine/threonine-protein kinase HT1-like (*G. max*).	Protein kinase superfamily protein.	At3g01490
**R12 JK845776**	XM_003529300	Protein E6-like (*G. max*).	Unknown protein.	At1g03820
**R13 JK845777**	XM_002325022	Predicted protein (*P. trichocarpa*).	Unknown protein.	At1g05205
**R14 JK845778**	XM_002275106	Two-component response regulator ARR1-like (*V. vinifera*).	ARR11 (response regulator 11).	At1g67710
**R15 JK845779**	XM_002326613	Predicted protein (*P. trichocarpa*).	Ribosomal protein L23/L15e family protein.	At4g16720
**R16 JK845780**	XM_002531538	Putative programmed cell death protein (*R. communis*).	RNA binding.	At3g11964
**R17 JK845781**	XM_002277369	CBL-interacting protein kinase 14 (CIPK14) mRNA (*V. vinifera*).	CIPK21 (CBL-interacting protein kinase 2.	At5g57630
**R18 JK845782**	BT089621	Clone SS0AEB28YD15 (*V. vinifera*).	NOI (RPM1-interacting protein 4 (RIN4) family protein).	At5g55850
**R19 JK845783**	XM_003540551	RNA-binding protein 38-like (*G. max*).	RNA-binding (RRM/RBD/RNP motifs) family protein.	At1g33470
**R20 JK845784**	XM_002520806	Putative alpha-galactosidase/alpha-n-acetylgalactosaminidase, mRNA (*R. communis*).	Melibiase family protein.	At3g56310
**R21 JK845785**	XM_002282890	Elicitor-responsive protein 3-like (*V. vinifera*).	C2 calcium/lipid-binding and GRAM domain containing Protein.	At5g50170
**R22 JK845786**	XM_002278821	Histone H3.3-like (*V. vinifera*).	Histone superfamily protein.	At5g10980
**R23 JK845787**	EU919653	Protein kinase haiku2 precursor (*P. dulcis*).	Leucine rich repeat (LRR) kinase IKU2 (HAIKU2).	At3g19700
**R24 JK845788**	EF147627	Clone WS0123_O22 unknown mRNA (*P. trichocarpa*).	Calmodulin binding protein (IQD14).	At2g43680
**R25 JK845789**	XM_002278482	Acyl-CoA synthetase short-chain family member 2 (ACSS2)(*V. vinifera*).	ACS (acetyl-CoA synthetase).	At5g36880
**R26 JK845790**	NM_001254526	Guanine nucleotide-binding protein subunit beta-like protein-like (*G. max*).	RACK1A (Transducin/WD40 repeat-like superfamily protein).	At1g18080
**R27 JK845791**	XM_002308368	Predicted protein (*P. trichocarpa*).	Putative endomembrane protein 70.	At5g25100
**R28 JK845792**	XM_002326378	1,4-alpha-glucan branching enzyme(*P. trichocarpa*).	SBE2.2 (Starch Branching Enzyme 2.2).	At5g03650
**R29 JK845793**	XM_002528381	Putative Rop guanine nucleotide exchange factor, mRNA (*R. communis*).	ROPGEF1 (Rho guanyl-nucleotide exchange factor).	At4g38430
**R30 JK845794**	XM_002282054	Ras-related protein Rab2BV-like (*V. vinifera*).	ATRABA2B (*Arabidops*is RAB GTPase homolog A2B).	At1g07410
**R31 JK845795**	EF640712	Pdbcs-L61 putative ethylene responsive protein (*P. dulcis*)	Adenine nucleotide alpha hydrolases-like superfamily protein.	At1g68300
**R32 JK845796**	XM_003538309	Uncharacterized protein LOC100783844 (*G. max*).	Unknown protein.	At1g47640
**R33 JK845797**	XM_002275701	Uncharacterized LOC100263264 (*V. vinifera*).	Protein of unknown function (DUF544).	At4g11860
**R34 JK845798**	XM_002267027	Putative vesicle-associated membrane protein 726-like(*V. vinifera*).	VAMP721 (Vesicle-Associated Membrane Protein 721).	At1g04750
**R35 JK845799**	XM_002512435	Putative syntaxin, mRNA (*R. communis*).	SYP43 (Syntaxin of plants 43).	At3g05710
**R36 JK845800**	XM_002274908	Probable protein phosphatase 2C 44-like (*V. vinifera*).	PIA1 (PP2C induced by AVRRPM1).	At2g20630
**R37 JK845801**	XM_002331064	Predicted protein (*P. trichocarpa*).	Lung seven transmembrane receptor family protein.	At5g18520
**R38 JK845802**	XM_002284976	DNA-binding protein S1FA2-like (*V. Vinifera*).	DNA-binding S1FA family protein.	At2g37120
**R39 JK845803**	AF336307	Auxin-repressed protein like-protein mRNA (*M. domestica*).	Dormancy/auxin associated family protein.	At2g33830
**R40 JK845804**	GU462127	Pectase lyase mRNA (*P. persica*).	Pectate lyase family protein.	At3g24670
**R41 JK845805**	GQ372916	Isolate PMG12 transposon Ty3-gypsy nonfunctional reverse transcriptase gene (*Prunus mume*).	Transposable element gene.	At2g10330
**Expression pattern: 111000**				
**R42 JK845806**	XM_002271609	Rae1-like protein At1g80670 (*V. vinifera*).	Transducin/WD40 repeat-like superfamily protein.	At1g80670
**Expression pattern: 011000**				
**R43 JK845807**	XM_003538456	Gamma-glutamyltranspeptidase 1-like (*G. max*).	GGT3 (Gamma-Glutamyl Transpeptidase 3).	At1g69820
**Expression pattern: 001000**				
**R44 JK845808**	XM_002320810	Hypothetical protein (*P. trichocarpa*).	ATAGP20 (Arabinogalactan Protein 20).	At3g61640

### Functional Classification of the Differential Expressed Unigenes

The query of the identified sequences on non-redundant databases (NCBI, ESTreeDB and TAIR) allowed to the attribution of 127 successfully BLAST hits. The functional identity of the isolated DEUs from the four clusters is depicted in Tables 2, 3, 4 and 5 taking into account the best (smallest output e-value) BLASTn hits for each cloned sequence.

**Table 4:** Functional identity and gene ontology description of the isolated unigenes corresponding to the “Other Group” (OG) unigenes.

Unigene Clone GenBank #	NCBI accession no.	NCBI and ESTreeDB functional annotation	TAIR Unigene functional annotation	*Arabidopsis* ortholog id.
**Expression pattern: 000001**				
**O1 JK845809**	XM_002285505	Beta-1,4-mannosyl-glycoprotein 4-beta-N-acetylglucosaminyltransferase-like (*V. vinifera*).	Beta-1,4-N-acetylglucosaminyltransferase family protein	At1g12990
**O2 JK845810**	JN559387	FK506-binding protein (*Fragaria x ananassa*).	FKBP12 (FK506-binding protein 12).	At5g64350
**O3 JK845811**	XM_002280485	Oligosaccharyl transferase STT3 protein-like (LTM1)(*V. vinifera*).	STT3A (Staurosporin and Temperature Sensitive 3-like A).	At5g19690
**O4 JK845812**	AK286496	cDNA clone: GMFL01-29-O19 (*G. max*).	Uncharacterized protein family SERF.	At3g24100
**Expression pattern: 100100**				
**O5 JK845813**	XM_002336575	Predicted protein, mRNA (*P. trichocarpa*).	Potential natural antisense gene.	At1g20691
**O6 JK845814**	XM_002274485	DNA-directed RNA polymerase 3, chloroplastic-like (*V. vinifera*).	Male Gametophyte defective 3, MGP3.	At1g68990
**O7 JK845815**	XM_002315378	Predicted protein, mRNA (*P. trichocarpa*).	Major facilitator superfamily protein.	At1g08900
**O8 JK845816**	XM_003592710	ABC transporter G family member (*M. truncatula).*	ABC-2 type transporter family protein.	At2g37360
**Expression pattern: 001010**				
**O9 JK845817**	AF319166	1-aminocyclopropane 1-carboxylic acid oxidase, mRNA (*P. persica*).	ACO2 (ACC oxidase 2).	At1g62380
**O10 JK845818**	AF298829	Putative protein disulfide-isomerase (PDI) mRNA(*P. avium*).	UNE5, MEE30, PDI11, ATPDI11 (thioredoxin family protein).	At2g47470
**Expression pattern: 001010**				
**O11 JK845819**	DQ424856	Chloroplast sequence (*V. vinifera*).	CLPP1 (plastid-encoded CLP P).	Atcg00670
**O12 JK845820**	XM_003590928	Hypothetical protein (MTR_1g080320) mRNA(*M. truncatula*).	Unknown protein.	At5g28960
**Expression pattern: 010010**				
**O13 JK845821**	NM_001251784	Protein kinase family protein (LOC100305405), mRNA (*G. max*).	Protein kinase protein with adeninenucleotide alpha hydrolases-like domain.	At1g77280

**Figure 2:**
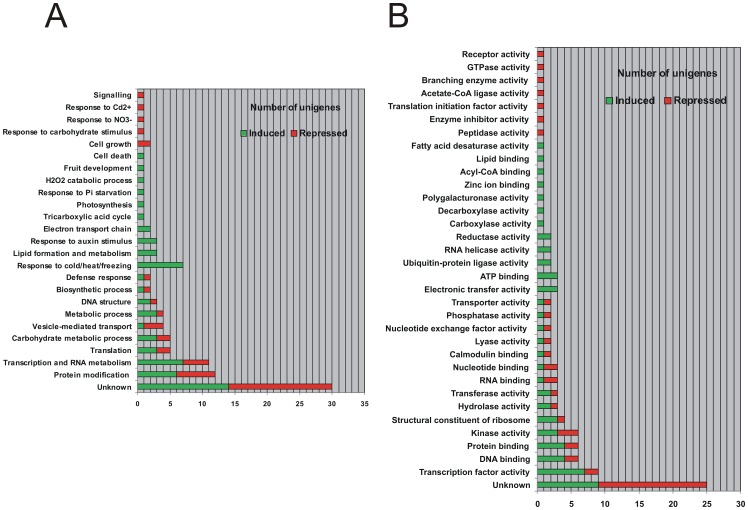
GO-term functional distribution of the heat-responsive unigenes. The top BLAST hits of the 105 differential expressed unigenes from the IG and RG were classified according to the GO term in biological processes (**A**) and molecular functions (**B**) vocabularies of their *Arabidopsis thaliana* orthologous.

The unigenes included in the IG and RG groups represent the heat-responsive genes in post-harvested peach fruits. Functional Gene-Ontology (GO) classification was performed to take an insight into the general biological processes and molecular functions that these heat-modified genes may have in the context of the post-harvest treatment. According to the histogram of frequency for the “biological process” classification, there are four high-representative groups of expression among the 25 total functional groups (Figure 2A). The most important one is the one with unknown function that it is composed of 30 unigenes (14 up-regulated and 16 down-regulated unigenes). This group is followed by two groups with fewer representatives, however, with higher frequency of appearance compared to the other 22 functional groups. These two clusters are involved in processes related to protein modification and transcription or RNA metabolism. Interestingly, the fourth most representative group (7 unigenes) is composed only of induced unigenes and gathered those genes involved in the response to cold, heat and freezing. Table S2 includes a table with detailed information about the unigenes that compose each functional group of expression, based on Gene Ontology (GO) annotation determined by the “biological process” term assigned to the most similar *Arabidopsis* protein.

**Table 5:** Functional identity and gene ontology description of the isolated unigenes corresponding to the “Harvest Genes” (HG) unigenes.

Unigene Clone GenBank #	NCBI accession no.	NCBI and ESTreeDB functional annotation.	TAIR Unigene functional annotation.	*Arabidopsis* ortholog id.
**Expression pattern: 100000**				
**H1 JK845822**	AC154901	BAC clone 82I18 (*P. persica*).	AP2/B3-like transcriptional factor family protein.	At5g25470
**H2 JK845823**	XM_002285442	Cysteinyl-tRNA synthetase-like (*V. vinifera*).	SYCO ARATH (Cysteinyl-tRNA synthetase,class Ia family protein).	At2g31170
**H3 JK845824**	CU226615	EST from severe drought-stressed leaves(*P. trichocarpa*).	ATHB-12 (homeobox 12).	At3g61890
**H4 JK845825**	XM_002312619	Predicted protein, mRNA (*P. trichocarpa*).	WIP4 (WIP domain protein 4).	At3g20880
**H5 JK845826**	EU915481	Ubiquitin-like protein 5 (UBL5) (*P. dulcis*) cultivar Fritz.	Ubiquitin-like superfamily protein.	At3g45180
**H6 JK845827**	XM_002516401	Putative map3k delta-1 protein kinase, mRNA(*R. communis*).	Protein kinase superfamily protein.	At5g11850
**H7 JK845828**	AJ243532	Metallothionein-like protein mRNA (*P. persica*).	MT2A (Metallothionein 2A).	At3g09390
**H8 JK845829**	XM_002307579	Predicted protein, mRNA (*P. trichocarpa*).	APG1 (S-adenosyl-L-methionine-dependent methyltransferases superfamily protein).	At3g63410
**H9 JK845830**	XM_002329638	Predicted protein, mRNA (*P. trichocarpa*).	TIM17-2 (Translocase Inner Membranesubunit 17-2).	At2g37410

Concerning the “molecular function” GO-terms there are 34 different functional clusters (Figure 2B). The most representative group is the one with “unknown molecular function”, having a total of 25 unigenes (9 up-regulated and 16 down-regulated). However, clusters related to transcription factor activity, DNA and protein binding and kinase activity are also highly represented in comparison with the other 29 functional groups. The unigene composition of each cluster, determined by the “molecular function” term assigned to the most similar *Arabidopsis* protein, is presented in a table in Table S3.

Besides, GO annotation for each peach heat responsive unigene was assessed by using the Blast2GO software. For some unigenes with unknown “biological process” or “molecular function” to the most similar *Arabidopsis* protein (Tables S2 and S3), some probable GO annotations were found, which are indicated in Table S4.

### Validation of the Differential Display Results through Quantitative Real-time PCR

**Figure 3:**
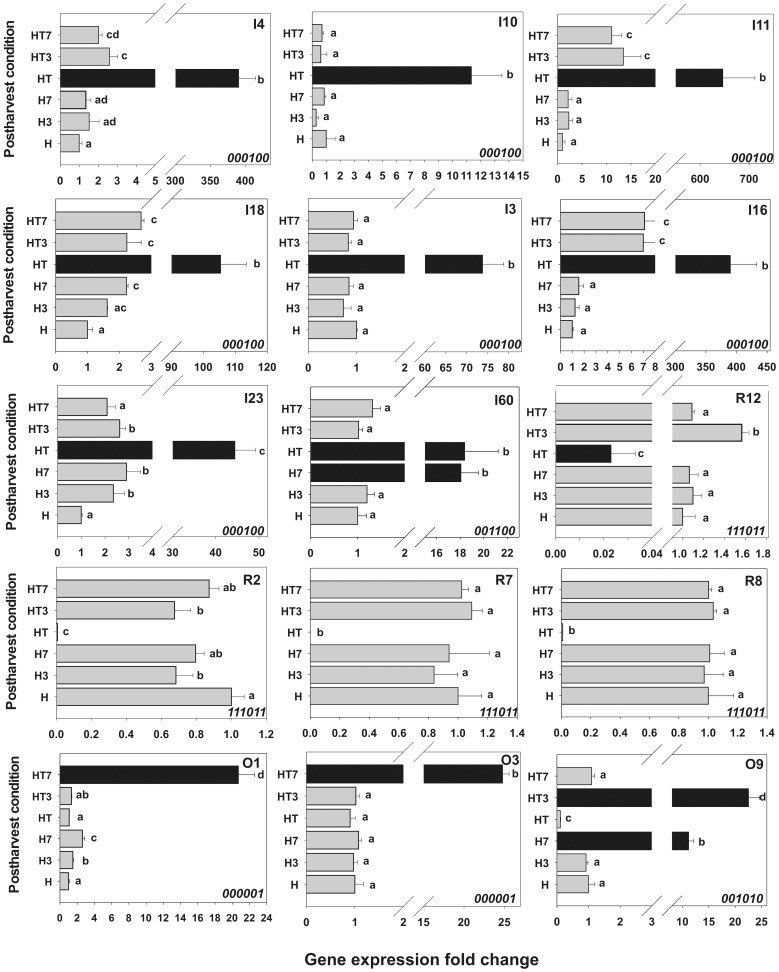
Validation of differentially expressed unigenes by qRT-PCR. The relative-to-harvest level of accumulation of the differentially expressed transcripts was determined in the different post-harvest conditions studied. Relative expression level indicated by black bars emphasized the presence-absent pattern of expression observed in the differential display experiments. Gene expression levels were normalized against *Arabidopsis thaliana rad50* (gb|AF168748.1|AF168748). Bars with at least one equal letter mean no statistically significant difference (α = 0.05). The expression pattern code (Table 1) of each unigene is indicated on the bottom right corner of each graph.

The transcriptional profiles obtained by the differential display technique were validated by quantifying the expression of 15 randomly chosen unigenes (Figure 3). Eight unigenes from the IG (I3, I4, I10, I11, I16, I18, I23 and I60), four from the RG (R2, R7, R8 and R12) and three from OG (O1, O3 and O9) were analyzed. Statistical analysis confirmed the significant differences in the relative level of expression of the unigenes chosen among the post-harvest samples included in the transcriptomic studies.

### Analysis of Heat Responsive Genes in Cold-treated Peach Fruits

Considering the protection against CI induced by HT, nearly 22% of the total heat responsive genes (Tables 2 and 3) were selected to analyze their responsiveness to cold in peach fruit. For this, a similar % of sequences from the Induced and Repressed Groups were selected randomly, in order to analyze the convergence of heat and cold signals in diverse biological processes. Thus, 23-heat responsive genes, 14 from the IG and 9 from the RG, were analyzed in peach fruit subjected to short-term cold storage for 3 (R3) or 5 days at 0°C (R5). Cold storage may affect post-translational and post-transcriptional processes, with changes in transcript expression being noticeable only after cold removal. Therefore, in addition to cold storage (R3 and R5), the response of these peach heat responsive genes was also evaluated after transferring the 5 day-cold stored fruits (R5) to 20°C for 2 days (R5+2).

**Figure 4:**
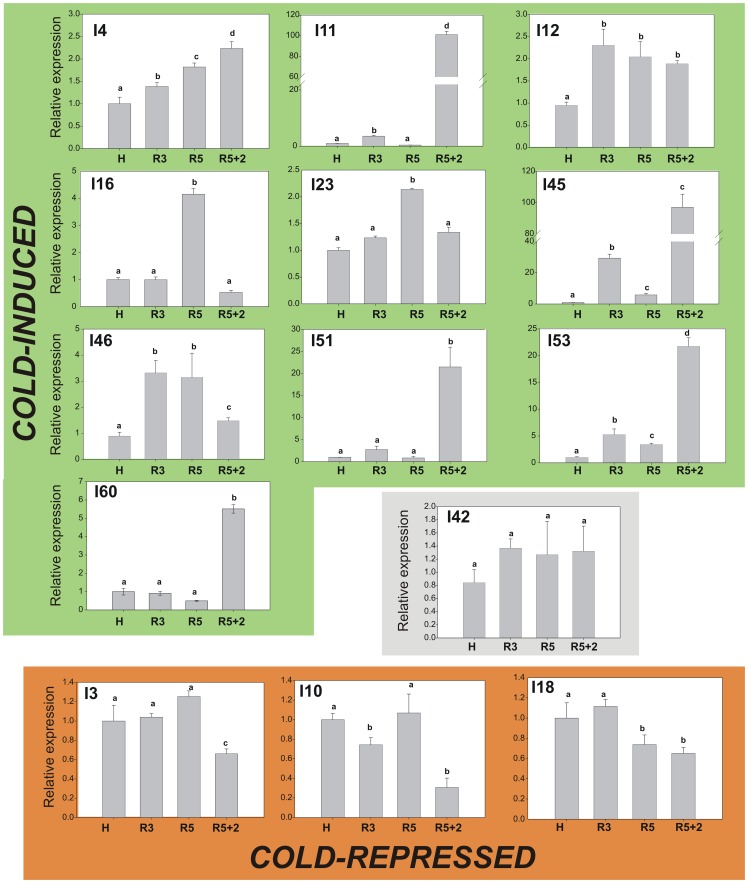
Heat-induced unigenes in cold-treated peach fruit. The relative-to-harvest (H) level of accumulation of 14 selected heat-induced transcripts was determined in peach fruit subjected to 3 (R3) or 5 days at 0°C (R5) followed by 2 days at 20°C (R5+2). Gene expression levels were normalized against *Arabidopsis thaliana rad50* (gb|AF168748.1|AF168748). Bars with at least one equal letter mean no statistically significant difference (α = 0.05). Cold-induced genes are grouped in green, cold-repressed transcripts in red and the transcript that was not modified by the cold treatment (I42) is in grey.

From the 14 transcripts selected from the heat-IG, 10 transcripts (I4, I11, I12, I16, I23, I45, I46, I51, I53 and I60) resulted induced by the cold treatment, either during storage at 0°C or after transfer to 20°C (Figure 4). Besides, only one transcript was not modified (I42), while the other 3 (I3, I10 and I18) resulted repressed by the cold treatment (Figure 4); thus displaying an opposite response to heat and cold.

**Figure 5:**
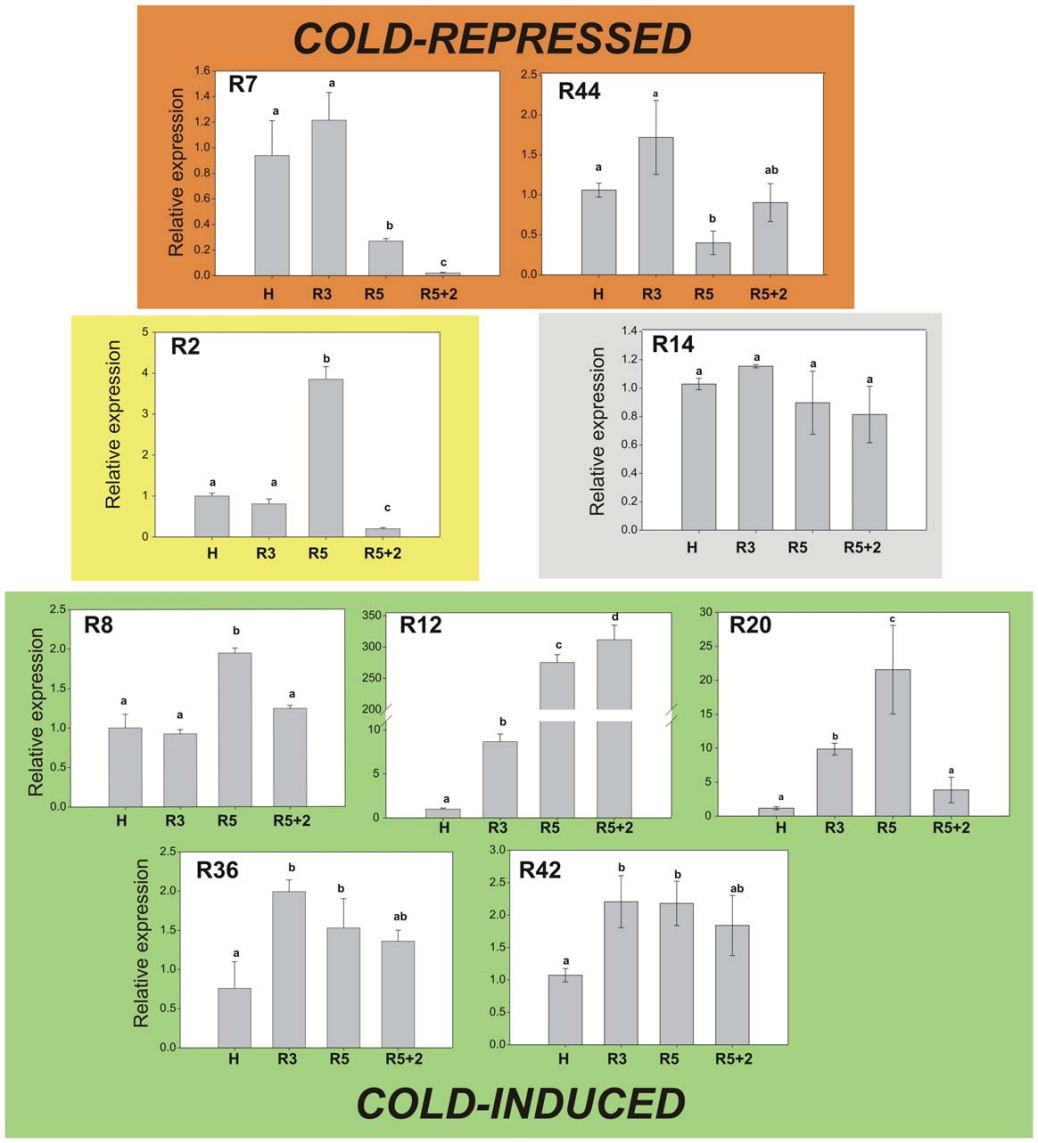
Heat-repressed unigenes in cold treated peach fruit. The relative-to-harvest (H) level of accumulation of 9 selected heat-repressed transcripts was determined in the peach fruit subjected to 3 (R3) or 5 days at 0°C (R5) followed by 2 days at 20°C (R5+2). Gene expression levels were normalized against *Arabidopsis thaliana rad50* (gb|AF168748.1|AF168748). Bars with at least one equal letter mean no statistically significant difference (α = 0.05). Cold-repressed transcripts are grouped in red and cold-induced transcripts in green. The transcript that was not modified by the cold treatment (R14) is in grey; while transcript R2, which resulted induced in R5 but repressed in R5+2, is in yellow.

On the other hand, from the 9 transcripts selected from the heat-RG, only one (R14) was not modified by the cold treatment (Figure 5). Two transcripts (R7 and R44) resulted repressed under cold storage at 0°C or after transfer to 20°C, while 5 transcripts (R8, R12, R20, R36 and R42) resulted increased by the cold treatment (Figure 5). One transcript (R2) resulted induced by cold storage for 5 days, but repressed when the fruits were transferred to 20°C for 2 days (Figure 5).

### *In silico* Analysis of Peach Heat Responsive Genes Regarding their Response to Cold Treatment in *Arabidopsis*

**Figure 6:**
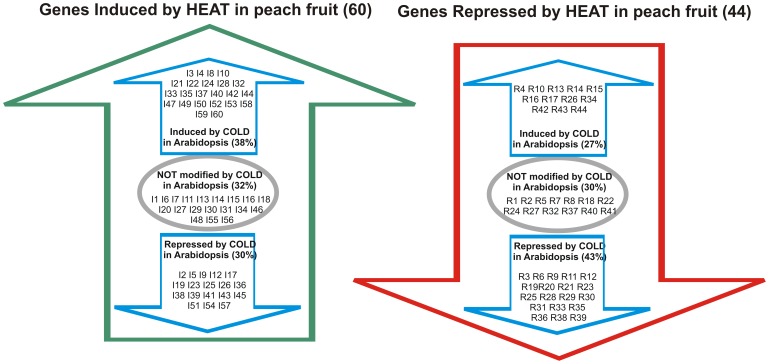
Classification of peach-heat-differentially expressed unigenes regarding their response to cold in *Arabidopsis*. *Arabidopsis* orthologs to the heat induced and repressed peach genes (Tables 2 and 3, respectively) were analyzed regarding their response to cold in *Arabidopsis* using the ColdArrayDB (http://cold.stanford.edu/cgi-bin/data.cgi). Within the induced and repressed peach genes, *Arabidopsis* orthologs with a fold-change higher than 1.5 in response to cold [33] are classified as induced or repressed, respectively. Genes with an absolute fold-change less than 1.5 are indicated as not modified by cold.

The heat-responsive genes in post-harvested peach fruits identified in the present work were also *in silico* analyzed regarding their response to cold in *Arabidopsis*. For this, *Arabidopsis* orthologs of the identified peach heat-responsive genes (IG and RG groups, Tables 2 and 3) were analyzed using the Cold Array Data Base [33]. Among the *Arabidopsis*’s orthologs to the peach heat responsive genes, a high percentage (nearly 70%, Figure 6) responds to cold (Figure 6). From these, nearly 40% of the *Arabidopsis*’s orthologs from both the IG and RG genes of peach fruit were regulated following the same trend by cold in *Arabidopsis* vegetative tissues (38% in the case of induced genes and 43% in the case of repressed genes; Figure 6). On the other hand, nearly 30% of *Arabidopsis*’s orthologs to the heat responsive genes from peach fruit had opposite response to cold in *Arabidopsis* vegetative tissues (up-regulated in peach but down-regulated in *Arabidopsis* and vice versa; Figure 6). Finally, nearly 30% of the *Arabidopsis*’s orthologs of heat responsive genes in peach did not respond to cold treatment in *Arabidopsis* vegetative tissue (Figure 6).

## Discussion

### Heat Treatment of Dixiland Peach Fruit Produces a Drastic Change in the Transcriptome with a High Impact on Protein and RNA Metabolism

Heat treatment is feasible for commercial application to post-harvest treatment of stone fruit and has shown to be sufficiently effective in controlling post-harvest diseases, reducing CI, maintaining fruit quality and preventing ripening in peach. In the present work, the application of HT (39°C during 3 days) on peach fruits after harvest generated a profound change in the transcriptome, with the identification of a total of 105 heat-responsive genes (Table 1). The functional classification of these genes indicated that those with unknown function are the most represented (nearly 30%, Figure 2), highlighting the importance of future detailed analysis of this group. The second most represented group (more than 20%) included genes involved in protein modification, transcription and RNA metabolism (Figure 2A). Thus, this group may be responsible for the signal transduction leading to the previously reported proteomic changes upon HT [14], [32]. Taking into account the generally low levels of expression of the proteins involved in signal transduction processes, changes in their expression were practically not detected in previous proteomic studies [14], [32].

Among this important group of genes, several transcriptional factors were up-regulated upon HT (Table 2). One of them, ZAT12 (I42, Table 2) has been proposed to be involved in cold acclimation and in oxidative stress signaling in *A. thaliana* [33], and it would be a converging node in different abiotic-stress networks [34]. Other up-regulated transcription factors, such as WRKY40 (I44, Table 2), IAA2 (I5, Table 2) or NF-YA4 (nuclear transcription factor Y, subunit A-4) (I52, Table 2), have been related to plant defense responses [35], auxin response [36], or in the modulation of ER stress-induced genes [37], respectively, and they may be involved in different biological processes that are modified upon HT of peach fruit. Genes that encode for a Dof-type zinc finger DNA-binding family protein (I55, Table 2) and an S-RNase (I57, Table 2) are also up-regulated by HT and related to transcription factors associated to light and defense responses [38].

Other up-regulated genes by HT probably involved in signal transduction include those that encode for helicases (I50, Table 2) and a RING finger helicase domain-containing protein (I22, Table 2), which *Arabidopsis*’s orthologs are both up-regulated by cold (Figure 6). A RNA-binding family protein (I29, Table 2) was also up-regulated by HT which, although it is classified as participating in RNA metabolism, it has not been established in which biological process it is involved (Table S2). Lastly, two genes encoding for zinc finger domain-containing proteins (I6 and I13, Table 2) are up-regulated by HT, although their biological function is unknown yet. In agreement with previous work that indicates that HT induces pathogen protection [30], several HT-induced genes identified in the present work have been involved in pathogen-response (Table 2).

### Heat and Cold Signals: Finding Common and Distinct Responses Related to Heat-induced Chilling Injury Protection

Previous transcriptional profiling analyses in peach fruit under cold storage have identified cold-responsive genes from peach mesocarp [3]–[5]. Among these cold-induced transcripts it is expected to find out those involved in the protection against the applied cold stress, but also those involved in the development of the CI symptoms, which include mealiness, browning, bleeding and/or loss of flavor [3]. In the present work, the transcriptomic modifications induced by the application of a treatment (HT) that induces CI protection was performed to find out genes involved in cold-protection, avoiding the detection of genes involved in the development of the CI-symptoms. Moreover, the differentially expressed genes upon HT were also analyzed under short-term cold treatment of peach fruit (Figure 4 and 5), to give an insight into the relationship between heat treatment and protection against CI. Moreover, it is expected that genes modified under short-term cold-storage are more probable related to cold response than to CI, since CI disorders appear after long-term cold storage. The results obtained indicate that more than 90% of the heat-responsive genes analyzed are also modulated by a short –cold exposure, from which nearly 60% show similar trend and nearly 40% the opposite response to heat and cold (Figure 4 and 5).

On the other hand, the functional characterization of the promoters of genes highly expressed in long term cold storage of peach showed heterologous regulation in *Arabidopsis* [39], which was related to the synteny conservation between the *Prunus* and *Arabidopsis* genomes [40]. With this in mind, peach heat-responsive genes identified in the present work were also clustered according to their response to cold in *Arabidopsis* (Figure 6). It is notable the high amount of genes (nearly 70%) that respond to both heat in peach and cold in *Arabidopsis*. From these genes, nearly 40% respond in the same way in both species and nearly 30% in an opposite manner (Figure 6).

The genes identified in the present work, that participate in the response to heat and cold in peach fruit, may be used in the future to design functional proofs for chilling injury susceptibility. For this, more studies on the relevance of these genes are needed, including the assessment of these genes in peach varieties with different susceptibility to chilling injury [4], [7], [41], [42]; and also in other model fruit species as *Solanum lycopersicum*, in which cold-response genes have also been identified [43].

Overall, although a deeper study of the genes identified in the present study is necessary, it is clear that the HT of peach induces a cold response that involves complex cellular processes. Plants sense cold stress through changes in membrane fluidity, protein and/or nucleic acid conformation leading to a complex transcriptional, post-transcriptional and post-translational regulation, which is critical to acquire cold acclimation [44]. In the present work, it is demonstrated that heat induces CI-protection in peach fruit by the modification of the expression of genes involved in protein modification, transcription and RNA metabolism, as well as a number of proteins with unknown function that need further analysis. The modification in the expression of these genes better prepares peach fruit for the subsequent cold-storage, avoiding the appearance of some CI symptoms.

### Conclusions

Acquired stress tolerance in plants is often a result of various stress-response mechanisms that act coordinately or synergistically to prevent cellular damage and to re-establish cellular homeostasis. In this work, the identification of genes modified by heat and cold tries to unravel the basis for the CI protection induced by HT in peach fruit, and highlights molecular candidates with technological application to improve shelf life features of peach fruit.

## Materials and Methods

### Plant Material

The plant material used for differential RNA display experiments consisted of *Prunus persica* (L.) Batsch cv Dixiland fruits grown at the Estación Experimental Agropecuaria INTA, San Pedro, Argentina and harvested in 2006 [14], [31]. Quantitative real-time PCR (qRT-PCR) experiments were performed using peach fruits harvested in 2008. Fruits were harvested when their flesh firmness reached 52.1±6.5 N, corresponding to approximately 90 days after bloom as described by [14]. Immediately after harvest, fruits were manually selected for uniformity of color, size and firmness and divided into two groups: one group was stored in a chamber at 20°C and 90% relative humidity for 7 days (control ripening group) and the other one was held in a chamber at 39±1°C and 90% relative humidity for 3 days (heat treatment) followed by 7 days at 20°C (heat treated ripening group). Collected samples consisted of representative mesocarp tissue from different fruits at six different post-harvest conditions from both groups: harvested fruits (designated as H); after 3 and 7 days in the chamber at 20°C (designated as H3 and H7, respectively); harvested fruits subjected to heat treatment during 3 days (designated as HT); 3-day heat-treated fruits after 3 and 7 days at 20°C (designated as HT3 and HT7, respectively) (Figure 1A). For the cold treatments, fruits were stored in a chamber at 0°C and 90% relative humidity for 3 (R3) or 5 days (R5) followed by 2 days at 20°C (R5+2). Flesh firmness, soluble solids, ground and pulp color and titratable acidity were determined as previously described in fresh fruits [31]. All the samples were immediately frozen in liquid nitrogen before storing at –80°C until they were used for RNA isolation.

### RNA Extraction

Total RNA, from the different studied samples of peach fruits (H, H3, H7, HT, HT3, HT7, R3, R5 and R5+2), was extracted and purified from 4 g of mesocarp tissue according to [45]. For all the samples, the RNA quality was determined by agarose electrophoresis. Additionally, the concentration and purity of each RNA preparation were determined spectrophotometrically. For quantitative real-time RT-PCR (qRT-PCR) studies total RNA was isolated and purified from 4 g of pooled mesocarpic tissue of three fruit in each condition using the method described above.

### Differential Display Assay

Differential display (DD) experiments were performed following the general protocol reported by [46] with some modifications. Eight µg of total RNA were used for the first-strand cDNA synthesis in 20 µl reactions containing 100 µM dNTP, 0.01 M DTT, 200 U of SuperScript II reverse transcriptase (Invitrogen) and 5 µM of one-base anchored oligonucleotide. The anchored oligonucleotides employed were named AA, AT, AC and AG and corresponded to sequences 5′ T_(16)_MN 3′, where M was degenerated A, C or G and N was A, C, G or T, respectively. Each of the four anchored primers was used for reverse transcription (RT) reactions for each sample analyzed. RT reactions were performed for 60 min at 38°C followed by a final enzyme inactivation step at 70°C for 15 min. Aliquots of 1 µl of each of the obtained cDNA sets (previously one-tenth diluted) were subjected to PCR containing the corresponding anchored primer (4 µM) and one of the RAPD-designed decameric random primers (0.8 µM; Table S5), 1X Taq activity buffer (Promega), 200 µM dNTPs, 2 U of GoTaq DNA polymerase enzyme (Promega) in a 20 µl final volume. In order to verify the absence of chromosomal DNA in the RNA preparations negative controls including a dilution of the non-reverse-transcribed total RNA were performed. All samples, including controls, were processed in duplicate. The cycle program for the PCR reactions consisted of an initial step of 3 min at 94°C, 40 cycles of 20 s at 94°C, 20 s at 38°C and 30 s at 72°C, followed by a final step of 5 min at 72°C. PCR products were mixed with equal amounts of denaturing loading buffer (95% (w/w) formamide, 10 mM NaOH, 0.05% (w/v) bromophenol, 0.05%(w/v) xylene cyanol) and treated for 3 min at 95°C and 7 µl each were separated on 0.4 mm thick, 5% (w/v) polyacrylamide/7.5 M urea/0.5X TBE (44 mM Tris–HCl, pH 8.0, 36 mM boric acid, 50 mM EDTA) sequencing gels (Electrophoresis System. DNA Sequencing System. FB-SEQ-3545, Fisher Scientific). Electrophoresis was performed at constant 60 W for 3–4 h. Separated DNA products were silver-stained following the DNA Silver Staining System procedure (Promega). The presence of a DNA band in the gel in a particular post-harvest condition indicated the expression of the transcript and it was assigned with the number of one (1) in the binary code used for classification (Table 1). On the contrary, the absence of a band indicated no expression and it was assigned with a zero (0) (Table 1). Differentially-expressed transcripts (DETs) were excised from the gel, eluted in a buffer containing 0.5 M ammonium acetate and 1 mM EDTA, pH 8.0, precipitated from the supernatant using ethanol and re-amplified employing the PCR conditions described above. The molecular weight of the DNA bands obtained ranged from 100 bp to 800 bp. A DNA band showing identical intensity among the compared samples was selected to be used as control of equal expression in quantitative PCR validation. Re-amplified DNA fragments were ligated into pGEM-T Easy Vector System (Promega) according to the manufacturer’s protocol and transformed into DH5α *Escherichia coli*. The transformed colonies were selected on LB agar plates supplemented with ampicillin, X-gal and IPTG. Selected clones from white colonies were grown overnight in LB growth media to performed multiple plasmid preparations by mean of the alkaline lysis method using “QIAprep Spin Miniprep Kit (QIAGEN)”. The presence and size of cDNA inserts were confirmed by restriction analysis using *Eco*RI enzyme. The recombinant plasmids were finally sequenced by using SP6- and T7 promoter sequencing primers at the Macrogen Inc (Korea) facility.

### Sequence Analysis and Functional Annotation

Nucleotide sequences retrieved by Macrogen Inc (Korea) were edited to remove vector sequences by using VecScreen analysis software (www.ncbi.nlm.nih.gov/VecScreen). All the cleaned sequences of DETs were analyzed using BLASTN on the basis of the existing annotation of non-redundant databases at: (1) the National Center of Biotechnology Information (NCBI; http://www.ncbi.nlm.nih.gov/pubmed/); (2) the *Prunus persica* EST collection at ESTreeDB database (http://www.itb.cnr.it/estree) [47]; (3) the TIGR transcript assembly database for all public *Prunus persica* and plant ESTs, or PlantTA (http://plantta.tigr.org) [48] and (4) The *Arabidopsis* Information Resource (http://www.arabidopsis.org). Sequences longer than 100 nucleotides were annotated as highly similar to the first BLAST hit when the e-value was lower than 1e^−5^. Next, over-represented DET sequences were organized as contigs and all the resulting analyzed DNA sequences were presented as unigenes (Table 1). Functional classification of the unigenes, identified by DD assay, was performed based on Gene Ontology (GO) annotation determined by the “biological process” and “molecular function” GO term assigned to the most similar *Arabidopsis* protein (Tables S2 and S3). Blast search and GO annotation of the heat responsive transcripts were also performed using Blast2GO software [49]. Table S4 shows GO identification obtained with this software for some unigenes with unknown “biological process” and “molecular function” GO annotation.

### Quantification of Transcript Levels Using Quantitative Real-time RT-PCR (qRT-PCR) Assay

The validation of the DET transcriptional patterns identified among the studied post-harvest samples was conducted using qRT-PCR amplification. Twelve percent of the identified unigenes were randomly selected for the qRT-PCR assays. Additionally, 23 unigenes were selected for analysis in peach fruits subjected to cold treatment. Gene specific primers (Table S6) were designed to produce amplicons of 140–250 bp length, with the aid of the web-based program “primer3” (http://www.frodo.wi.mit.edu/cgi-bin/primer3/primer3_www.cgi). A sequence homologous (e-value = 7e^−44^) to the *A. thaliana rad50* (gb|AF168748.1|AF168748), coding for the DNA repair-recombination protein (RAD50), was used as endogenous reference gene, equally expressed among the studied samples. The PCR primers designed for the reference gene were 5′ TGAGCCTCTTCCAAATGCTT 3′ for the forward, and 5′ ACGGTGGCACAGTCTAAAGG 3′ for the reverse, generating a product of 141 bp. First-strand cDNAs, used in the corresponding validation assays, were synthesized with MMLV-reverse transcriptase (Promega) as indicated by the manufacturers, employing 3 µg of total RNA (extracted as described above) and oligo(dT). qRT-PCR assays were prepared in a final volume of 20 µl containing 1X Taq activity buffer (Promega), 200 µM dNTPs, 2.5 mM MgCl_2_, 0.8 U of GoTaq DNA polymerase enzyme (Promega), 0.5 µM of each primer (Table S6), 0.5X SYBRGreen I (Invitrogen), and 1 µl of a ten-fold dilution of cDNA each. qRT-PCR controls were performed including dilution of non-reverse-transcribed total RNA in order to ensure RNA preparations were free of DNA contamination. The cycling parameters were as follows: an initial denaturation step at 94°C for 2 min; 40 cycles of 96°C for 10 s; 58°C for 15 s; 72°C for 1 min, and 78°C for 1 s to detect fluorescence, and final elongation step at 72°C for 10 min. The high reading plate temperature of 78°C was set to avoid the influence of primer dimers to fluorescence detection. Melting curves for each qRT-PCR reaction were constructed for specific detection of the amplified product by increasing the temperature from 65°C to 98°C. The specificity of the qRT-PCR reactions was also confirmed by analysing the uniqueness of the resulting amplicons separated in a 2% (w/v) non-denatured agarose gel. The Ct values were assayed three times for each of the samples employing at least two independent (three-fruit pooled each) biological samples for each analyzed post-harvest condition. All the reactions were performed in an iCycler iQ detection system (equipped with a 48 well plate) with the Optical System Software version 3.0a (Bio-Rad). All quantifications were normalized to the identified endogenous reference gene (*rad50*) used as housekeeping gene and amplified in the same conditions. Relative gene expression was calculated using the Comparative 2^−ΔΔCt^ method [50]. Statistical analyses were carried out using one-way analysis of variance (ANOVA). Significant differences were assessed by the Bonferroni test (α = 0.05) using the Sigma Stat Package.

### Analysis of Heat-differential Expressed Genes in the *Arabidopsis* Cold Array Data Base

*Arabidopsis* orthologs of the identified heat-responsive genes (Tables 2 and 3) were analyzed using the *Arabidopsis* Cold Array Data Base (ColdArrayDB, http://cold.stanford.edu/cgi-bin/data.cgi). The identified genes with a fold-change higher than 1.5 on the Affymetrix 23 K array [33] were classified as induced or repressed by cold. The experiment of plates containing plants transferred to 4°C and harvested after various time periods was considered for the analysis (ColdArrayDB, http://cold.stanford.edu/cgi-bin/data.cgi).

## Supporting Information

Table S1 **Principal quality parameters of Dixiland peach fruits at harvest (H).**; after 3 or 7 days along the normal ripening process at 20°C (H3 and H7, respectively); after the application of a heat treatment of 39°C for 3 days (HT) followed by 3 or 7 days at 20°C (HT3 and HT7, respectively) and after 3 or 5 days at 0°C following the harvest (R3 and R5, respectively).
(PDF)

Table S2 **Unigene classification according to their GO “biological process” term assigned to the most similar** ***Arabidopsis*** **protein.**
(PDF)

Table S3 **Unigene classification according to their GO “molecular function” term assigned to the most similar** ***Arabidopsis*** **protein.**
(PDF)

Table S4 **GO Identification, obtained using the Blast2GO software** [**49**] **, for some unigenes with unknown “biological process” and “molecular function” GO annotation (Tables S2 and S3).** F: Molecular Function; P: Biological Process.
(PDF)

Table S5 **Decameric primers employed for the Differential Display analysis.**
(PDF)

Table S6 **Primers employed for qRT-PCR validation.**
(PDF)

## References

[1] Lurie S, Crisosto CH (2005) Chilling injury in peach and nectarine. Postharvest Biol Technol 37: 195–208.

[2] González-Agüero M, Pavez L, Ibáñez F, Pacheco I, Campos-Vargas R, et al (2008) Identification of woolliness response genes in peach fruit after post-harvest treatments. J Exp Bot 59: 1973–1986. 18453640 10.1093/jxb/ern069 PMC2413281

[3] Ogundiwin EA, Martí C, Forment J, Pons C, Granell A, et al (2008) Development of ChillPeach genomic tools and identification of cold-responsive genes in peach fruit. Plant Mol Biol 68: 379–397. 18661259 10.1007/s11103-008-9378-5

[4] Falara V, Manganaris GA, Ziliotto F, Manganaris A, Bonghi C, et al (2011) A ß- D -xylosidase and a PR-4B precursor identified as genes accounting for differences in peach cold storage tolerance. Funct Integr Genomics 11: 357–368. 21221699 10.1007/s10142-010-0204-1

[5] Vizoso P, Meisel LA, Tittarelli A, Latorre M, Saba J, et al (2009) Comparative EST transcript profiling of peach fruits under different post-harvest conditions reveals candidate genes associated with peach fruit quality. BMC Genomics 10: 423. 19744325 10.1186/1471-2164-10-423 PMC2748099

[6] Brummell DA, Dal Cin V, Lurie S, Crisosto CH, Labavitch JM (2004) Cell wall metabolism during the development of chilling injury in cold-stored peach fruit: association of mealiness with arrested disassembly of cell wall pectins. J Exp Bot 55: 2041–2052. 15310820 10.1093/jxb/erh228

[7] Dagar A, Friedman H, Lurie S (2010) Thaumatin-like proteins and their possible role in protection against chilling injury in peach fruit. Postharvest Biol Technol 57: 77–85.

[8] Nilo R, Saffie C, Lilley K, Baeza-Yates R, Cambiazo V, et al (2010) Proteomic analysis of peach fruit mesocarp softening and chilling injury using difference gel electrophoresis (DIGE). BMC Genomics 11: 43. 20082721 10.1186/1471-2164-11-43 PMC2822761

[9] Zhang C, Ding Z, Xu X, Wang Q, Qin G, et al (2010) Crucial roles of membrane stability and its related proteins in the tolerance of peach fruit to chilling injury. Amino acids 39: 181–94. 20091071 10.1007/s00726-009-0397-6

[10] Zhang C, Tian S (2009) Crucial contribution of membrane lipids’ unsaturation to acquisition of chilling-tolerance in peach fruit stored at 0°C. Food Chem 115: 405–411.

[11] Zhang C, Tian S (2010) Peach fruit acquired tolerance to low temperature stress by accumulation of linolenic acid and N-acylphosphatidylethanolamine in plasma membrane. Food Chem 120: 864–872.

[12] Wang L, Chen S, Kong W, Li S, Archbold DD (2006) Salicylic acid pretreatment alleviates chilling injury and affects the antioxidant system and heat shock proteins of peaches during cold storage. Postharvest Biol Technol 41: 244–251.

[13] Jin P, Zheng Y, Tang S, Rui H, Wang CY (2009) A combination of hot air and methyl jasmonate vapor treatment alleviates chilling injury of peach fruit. Postharvest Biol Technol 52: 24–29.

[14] Lara MV, Borsani J, Budde CO, Lauxmann MA, Lombardo VA, et al (2009) Biochemical and proteomic analysis of “Dixiland” peach fruit (*Prunus persica*) upon heat treatment. J Exp Bot 60: 4315–4333. 19734260 10.1093/jxb/erp267

[15] Lara MV, Budde CO, Porrini L, Borsani J, Murray R, et al (2011) Peach (*Prunus persica*) fruit response to anoxia: Reversible ripening delay and biochemical changes. Plant Cell Physiol 52: 392–403. 21186173 10.1093/pcp/pcq200

[16] Cao S, Hu Z, Zheng Y, Lu B (2010) Synergistic effect of heat treatment and salicylic acid on alleviating internal browning in cold-stored peach fruit. Postharvest Biol Technol 58: 93–97.

[17] Pegoraro C, Zanuzo MR, Chaves FC, Brackmann A, Girardi CL, et al (2010) Physiological and molecular changes associated with prevention of woolliness in peach following pre-harvest application of gibberellic acid. Postharvest Biol Technol 57: 19–26.

[18] Pegoraro C, Chaves FC, Dal Cero J, Girardi CL, Rombaldi CV (2011) Effects of pre-harvest gibberellic acid spraying on gene transcript accumulation during peach fruit development. Plant Growth Regul 65: 231–237.

[19] Yang A, Cao S, Yang Z, Cai Y, Zheng Y (2011) γ-Aminobutyric acid treatment reduces chilling injury and activates the defence response of peach fruit. Food Chem 129: 1619–1622.

[20] Yang Z, Cao S, Zheng Y, Jiang Y (2012) Combined salicylic acid and ultrasound treatments for reducing the chilling injury on peach fruit. J Agr Food Chem 60: 1209–1212. 22229406 10.1021/jf2041164

[21] Sabehat A, Lurie S, Weiss D (1998) Expression of small heat shock proteins at low temperature: a possible role in protecting against chilling injuries. Plant Physiol 117: 651–658. 9625718 10.1104/pp.117.2.651 PMC34985

[22] Sabehat A, Weiss D, Lurie S (1996) The correlation between Heat-Shock Protein accumulation and persistence and chilling tolerance in tomato fruit. Plant Physiol 110: 531–537. 8742333 10.1104/pp.110.2.531 PMC157748

[23] Lurie S (1998) Postharvest heat treatments. Postharvest Biol Technol 14: 257–269.

[24] Lurie S (2006) The effect of high temperature treatment on quality of fruits and vegetables. Acta Horticulturae 712: 165–173.

[25] Paull RE, Chen NJ (2000) Heat treatment and fruit ripening. Postharvest Biol Technol 21: 21–37. 10.1016/s0925-5214(98)00101-x 11543413

[26] Ferguson IB, Ben-Yehoshua S, Mitcham EJ, Mc Donald RE, Lurie S (2000) Postharvest heat treatments. Introduction and workshop summary. Postharvest Biol Technol 21: 1–6.

[27] Sanchez-Ballesta MT, Lluch Y, Gosalbes MJ, Zacarias L, Granell A, et al (2003) A survey of genes differentially expressed during long-term heat-induced chilling tolerance in citrus fruit. Planta 218: 65–70. 13680227 10.1007/s00425-003-1086-4

[28] Sapitnitskaya M, Maul P, McCollum GT, Guy CL, Weiss B, et al (2006) Postharvest heat and conditioning treatments activate different molecular responses and reduce chilling injuries in grapefruit. J Exp Bot 57: 2943–2953. 16908505 10.1093/jxb/erl055

[29] Polenta GA, Calvete JJ, González CB (2007) Isolation and characterization of the main small heat shock protein induced in tomato pericarp by thermal treatment. FEBS J 274: 6447–6455. 18021250 10.1111/j.1742-4658.2007.06162.x

[30] Liu J, Sui Y, Wisniewski M, Droby S, Tian S, et al (2012) Effect of heat treatment on inhibition of *Monilinia fructicola* and induction of disease resistance in peach fruit. Postharvest Biol Technol 65: 61–68.

[31] Borsani J, Budde CO, Porrini L, Lauxmann MA, Lombardo VA, et al (2009) Carbon metabolism of peach fruit after harvest: changes in enzymes involved in organic acid and sugar level modifications. J Exp Bot 60: 1823–1837. 19264753 10.1093/jxb/erp055

[32] Zhang L, Yu Z, Jiang L, Jiang J, Luo H, et al (2011) Effect of post-harvest heat treatment on proteome change of peach fruit during ripening. J Proteomics 74: 1135–1149. 21550427 10.1016/j.jprot.2011.04.012

[33] Vogel JT, Zarka DG, Van Buskirk HA, Fowler SG, Thomashow MF (2005) Roles of the CBF2 and ZAT12 transcription factors in configuring the low temperature transcriptome of Arabidopsis. Plant J 41: 195–211. 15634197 10.1111/j.1365-313X.2004.02288.x

[34] Davletova S, Schlauch K, Coutu J, Mittler R (2005) The zinc-finger protein zat12 plays a central role in reactive oxygen and abiotic stress signaling in Arabidopsis. Plant Physiol 139: 847–856. 16183833 10.1104/pp.105.068254 PMC1256000

[35] Xu X, Chen Ch, Fan B, Chen Z (2006) Physical and functional interactions between pathogen-induced Arabidopsis WRKY18, WRKY40, and WRKY60 transcription factors. Plant Cell 18: 1310–1326. 16603654 10.1105/tpc.105.037523 PMC1456877

[36] Trainotti L, Tadiello A, Casadoro G (2007) The involvement of auxin in the ripening of climacteric fruits comes of age: the hormone plays a role of its own and has an intense interplay with ethylene in ripening peaches. J Exp Bot 58: 3299–3308. 17925301 10.1093/jxb/erm178

[37] Liu JX, Howell SH (2010) bZIP28 and NF-Y transcription factors are activated by ER stress and assemble into a transcriptional complex to regulate stress response genes in Arabidopsis. Plant Cell 22: 782–796. 20207753 10.1105/tpc.109.072173 PMC2861475

[38] Yanagisawa S (2002) The Dof family of plant transcription factors. Trends Plant Sci. 7: 555–60. 10.1016/s1360-1385(02)02362-2 12475498

[39] Tittarelli A, Santiago M, Morales A, Meisel LA, Silva H (2009) Isolation and functional characterization of cold-regulated promoters, by digitally identifying peach fruit cold-induced genes from a large EST dataset. BMC Plant Biol 9: 121. 19772651 10.1186/1471-2229-9-121 PMC2754992

[40] Jung J, Dorrie M, Staton M, Cho I, Zhebentyayeva T, et al (2006) Synteny conservation between the Prunus and both the present and ancestral Arabidopsis genomes. BMC Genomics 7: 81. 16615871 10.1186/1471-2164-7-81 PMC1479338

[41] Dagar A, Weksler A, Friedman H, Ogundiwin EA, Crisosto CH, et al (2011) Comparing ripening and storage characteristics of `Oded´ peach and its nectarine mutant `Yuvaĺ. Postharvest Biol Technol 60: 1–6.

[42] Dagar A, Pons Puig C, Ibanez CM, Ziliotto F, Bonghi C, et al.. (2012) Comparative transcript profiling of a peach and its nectarine mutant at harvest reveals differences in gene expression related to storability. Tree Gen & Gen DOI 10.1007/s11295-012-0549-9.

[43] Rugkong A, McQuinnc R, Giovannoni JJ, Rosee JKC, Watkins CB (2011) Expression of ripening-related genes in cold-stored tomato fruit. Postharvest Biol Technol 61: 1–14.

[44] Chinnusamy V, Zhu J, Zhu J-K (2007) Cold stress regulation of gene expression in plants. Trends Plant Sci 12: 444–451. 17855156 10.1016/j.tplants.2007.07.002

[45] Meisel L, Fonseca B, González S, Baeza-Yates R, Cambiazo B, et al (2005) A rapid and efficient method for purifying high quality total RNA from peaches (Prunus persica) for functional genomics analysis. Biol Res 38: 83–88. 15977413 10.4067/s0716-97602005000100010

[46] Liang P, Pardee AB (1998) Differential display. A general protocol. Molecular Biotechnol 3: 261–267. 10.1007/BF02740847 9951706

[47] Lazzari B, Caprera A, Vecchietti A, Merelli I, Barale F, et al (2008) Version VI of the ESTree db: an improved tool for peach transcriptome analysis. BMC Bioinformatics 9: S9. 10.1186/1471-2105-9-S2-S9 PMC2323672 18387211

[48] Childs KL, Hamilton JP, Zhu W, Ly E, Cheung F, et al (2007) The TIGR Plant Transcript Assemblies database. Nucleic Acids Res 35: D846–851. 17088284 10.1093/nar/gkl785 PMC1669722

[49] Conesa A, Götz S, García-Gómez JM, Terol J, Talón M, et al (2005) Blast2GO: a universal tool for annotation, visualization and analysis in functional genomics research. Bioinformatics 21: 3674–3676. 16081474 10.1093/bioinformatics/bti610

[50] Livak KJ, Schmittgen TD (2001) Analysis of relative gene expression data using real-time quantitative PCR and the 2−ΔΔCt. Methods 25: 402–408. 11846609 10.1006/meth.2001.1262

