# Supplementary material for: Transcriptomic Profiling during the Post-Harvest of Heat-Treated Dixiland Prunus persica Fruits: Common and Distinct Response to Heat and Cold
Source: PLoS One. 2012 Dec 6;7(12):e51052. doi: 10.1371/journal.pone.0051052 (PMC3516522; doi:10.1371/journal.pone.0051052)
Supplement: Table S1 — Principal quality parameters of Dixiland peach fruits at harvest (H).; after 3 or 7 days along the normal ripening process at 20°C (H3 and H7, respectively); after the application of a heat treatment of 39°C for 3 days (HT) followed by 3 or 7 days at 20°C (HT3 and HT7, respectively) and after 3 or 5 days at 0°C following the harvest (R3 and R5, respectively). (PDF) [file pone.0051052.s001.pdf]

Table S1. Principal quality parameters of Dixiland peach fruits at harvest (H); after 3 or 7 days along the normal ripening process at 20°C (H3 and H7, respectively); after the application of a heat treatment of 39°C for 3 days (HT) followed by 3 or 7 days at 20°C (HT3 and HT7, respectively) and after 3 or 5 days at 0ºC following the harvest (R3 and R5, respectively).

				Sample				
**Parameter**	**H**	**H3**	**H7**	**HT**	**HT3**	**HT7**	**R3**	**R5**
**Firmness (N)**	52.1 ± 6.5[a]	40. 2 ± 9.1[b]	11.9 ± 2.9[c]	53.0 ± 7.7[a]	4.3 ± 1.2[d]	4.5 ± 1.0[d]	51.9 ± 5.8[a]	50.9 ± 6.1[a]
**Ground Colour**								
***H* value**	103.1 ± 2.3[a]	99.9 ± 2.2[a]	92.6 ± 2.6[b]	92.4 ± 1.9[b]	73.6 ± 6.0[c]	72.8 ± 5.6[c]	101.2 ± 2.5[a]	100.1 ± 3.3[a]
**Pulp colour**								
***H* value**	97.0 ± 2.0[a]	95.8 ± 1.5[a]	92.5 ± 1.4[b]	93.3 ±1.9[b]	85.2± 2.5[c]	79.2± 1.9[d]	96.8 ± 2.5[a]	96.0 ± 2.0[a]
**Soluble solids (ºBrix)**	10.6 ± 0.9[ab]	11.6 ± 1.3[a]	11.0 ± 1.1[ab]	10.3 ± 0.8[b]	11.4 ± 1.2[ab]	11.5 ± 0.9[ab]	10.4 ± 0.9[ab]	10.8 ± 1.5[ab]
**Acidity [H^+] (M)**	0.14 ± 0.01[a]	0.15 ± 0.01[a]	0.14 ± 0.01[a]	0.08 ± 0.01[b]	0.10 ± 0.01[c]	0.11 ± 0.02[c]	0.13 ± 0.02[a]	0.13 ± 0.03[a]

Different letters within each parameter indicate statistically significant differences.
