# Supplementary material for: Transcriptomic Profiling during the Post-Harvest of Heat-Treated Dixiland Prunus persica Fruits: Common and Distinct Response to Heat and Cold
Source: PLoS One. 2012 Dec 6;7(12):e51052. doi: 10.1371/journal.pone.0051052 (PMC3516522; doi:10.1371/journal.pone.0051052)
Supplement: Table S2 — Unigene classification according to their GO “biological process” term assigned to the most similar Arabidopsis protein. (PDF) [file pone.0051052.s002.pdf]

Table S2. Unigene classification according to their GO "biological process" term assigned to the most similar *Arabidopsis* protein

Biological process	Induced unigenes	Repressed unigenes
Unknown	I6; I13; I15; I16; I17; I19; I20; I21; I29; I38; I47; I50; I56; I59	R5; R6; R8; R12; R13; R19; R21; R27; R31; R32; R33; R34; R37; R39; R41; R44
Protein modification	I9; I26; I27; I31; I36; I49	R4; R9; R11; R17; R23; R42
Transcription and RNA metabolism	I1; I2; I24; I44; I52; I55; I57;	R7; R14; R16; R38
Translation	I3; I21; I35	R10; R15
Carbohydrate metabolic process	I30; I32; I40	R20; R28
Vesicle-mediated transport	I43	R30; R34; R35
Metabolic process	I4; I11; I58	R25
DNA structure	I22; I48	R22
Biosynthetic process	I8	R2
Defense response	I53	R3
Response to cold, feezing and heat	I12; I34; I39; I42; I46; I51; I60	none
Lipid formation and metabolism	I37; I41; I45	none
Response to auxin stimulus	I2; I5; I23	none
Electron transport chain	I10; I14	none
Tricarboxylic acid cycle	I18	none
Photosynthesis	I28	none
Response to Pi starvation	I54	none
H_2O_2 catabolic process	I7	none
Fruit development	I33	none
Cell death	I25	none
Cell growth	none	R1; R29
Response to carbohydrate stimulus	none	R39
Response to nitrate	none	R18
Response to cadmium ion	none	R26
Signalling	none	R24
