# Supplementary material for: Transcriptomic Profiling during the Post-Harvest of Heat-Treated Dixiland Prunus persica Fruits: Common and Distinct Response to Heat and Cold
Source: PLoS One. 2012 Dec 6;7(12):e51052. doi: 10.1371/journal.pone.0051052 (PMC3516522; doi:10.1371/journal.pone.0051052)
Supplement: Table S3 — Unigene classification according to their GO “molecular function” term assigned to the most similar Arabidopsis protein. (PDF) [file pone.0051052.s003.pdf]

Table S3. Unigene classification according to their GO "molecular function" term assigned to the most similar *Arabidopsis* protein.

Molecular function	Induced unigenes	Repressed unigenes
Unknown	I13; I15; I17; I27; I28; I37; I38; I43; I56	R5; R7; R8; R12; R13; R18; R21; R27; R31; R32; R33; R34; R37; R39; R41; R44
Transcription factor activity	I1; I2; I42; I44; I52; I55; I57	R1; R14
DNA binding	I16; I22; I24; I48	R22; R38
Protein binding	I26; I41; I53; I54	R3; R9
Kinase activity	I20; I31; I49	R11; R17; R23
Structural constituent of ribosome	I3; I21; I35;	R15
Hydrolase activity	I30; I33	R20
Transferase activity	I32; I59	R43
RNA binding	I29	R16; R19
Nucleotide binding	I47	R26; R42
Calmodulin binding	I25	R24
Lyase activity	I11	R40
Nucleotide exchange factor activity	I23	R29
Phosphatase activity	I58	R36
Transporter activity	I60	R2
Electronic transfer activity	I10; I12; I14	none
ATP binding	I34; I39; I53	none
Ubiquitin-protein ligase activity	I9; I36	none
RNA helicase activity	I46; I50	none
Reductase activity	I7; I8	none
Carboxilase activity	I18	none
Decarboxilase activity	I4	none
Poligalacturonase activity	I40	none
Zinc ion binding	I6	none
Acyl-CoA binding	I51	none
Lipid binding	I19	none
Fatty acid desaturase activity	I45	none
Peptidase activity	none	R4
Enzyme inhibitor activity	none	R6
Translation initiation factor activity	none	R10
Acetate-CoA ligase activity	none	R25
Branching enzyme activity	none	R28

GTPase activity	none	R30
Receptor activity	none	R35
