# Supplementary material for: Transcriptomic Profiling during the Post-Harvest of Heat-Treated Dixiland Prunus persica Fruits: Common and Distinct Response to Heat and Cold
Source: PLoS One. 2012 Dec 6;7(12):e51052. doi: 10.1371/journal.pone.0051052 (PMC3516522; doi:10.1371/journal.pone.0051052)
Supplement: Table S4 — GO Identification, obtained using the Blast2GO software [49] , for some unigenes with unknown “biological process” and “molecular function” GO annotation (Tables S2 and S3). F: Molecular Function; P: Biological Process. (PDF) [file pone.0051052.s004.pdf]

Table S4. GO Identification, obtained using the Blast2GO software [49], for some unigenes with unknown "biological process" and "molecular function" GO annotation (Additional Files 2 and 3). F: Molecular Function; P: Biological Process

**Unigene**	**GO Id**
I6	F: Zinc ion binding<br>P: Vesicle-mediated transport
I14	F: electron carrier activity<br>P: photorespiration; proteasome core complex assembly; response to misfolded protein; ubiquitin-dependent protein catabolic process
I15	P: vernalization response
I21	F: structural constituent of ribosome<br>P: translation
I29	F: nucleic acid binding; RNA binding
I38	F: transferring glycosyl groups<br>P: microtubule nucleation
I50	F: helicase activity, RNA binding; nucleic acid binding; ATP-dependent helicase activity; microtubule cytoskeleton organization<br>P: cytokinesis by cell plate formation; protein glycosylation
I56	F: hydrolase activity; lyase activity
I59	F: transferase activity, transferring glycosyl groups
R6	F: heme binding; iron ion binding; oxidoreductase activity, metal ion binding; monooxygenase activity<br>P: negative regulation of catalytic activity; oxidation-reduction process
R13	P: photorespiration
R18	P: Golgi vesicle transport; cellular membrane fusion; response to nitrate
R31	P: response to stress; response to molecule of fungal origin
R32	P: vesicle-mediated transport
R34	P:vesicle-mediated transport; transport; cell plate formation involved in plant-type cell wall biogenesis
R39	F: lyase activity<br>P: auxin mediated signaling pathway
R41	F: RNA binding; nucleic acid binding; ribonuclease H activity<br>P: DNA integration; RNA-dependent DNA replication
R44	P: tryptophan catabolic process; indoleacetic acid biosynthetic process
