# Supplementary material for: Transcriptomic Profiling during the Post-Harvest of Heat-Treated Dixiland Prunus persica Fruits: Common and Distinct Response to Heat and Cold
Source: PLoS One. 2012 Dec 6;7(12):e51052. doi: 10.1371/journal.pone.0051052 (PMC3516522; doi:10.1371/journal.pone.0051052)
Supplement: Table S5 — Decameric primers employed for the Differential Display analysis. (PDF) [file pone.0051052.s005.pdf]

Table S5. Decameric primers employed for the Differential Display analysis

**Primer**	**Sequence**	**Melting temperature (°C)**
A1	5’CCCAAGGTCC3’	34
A2	5’ GGTGCGGGAA3’	34
A3	5’ AAGACCCCTC3’	32
A4	5’CTTCACCCGA3’	32
A5	5’CACCAGGTGA3’	32
A6	5’GAGTCTCAGG3’	32
A7	5’CCCGATTCGG3’	34
A8	5’ACGCACAACC3’	32
A9	5’CTAATGCCGT3’	30
A10	5’ACGGCGTATG3’	32
B1	5’TCGAAGTCCT3’	30
B2	5’GCATGTCAGA3’	30
B3	5’ACTTCGACAA3’	32
B4	5’TGCCATCAGT3’	30
B5	5’GCGCTCACGC3’	36
B6	5’GTGACATGCC3’	32
B7	5’AGATCGAGCC3’	32
B8	5’TCACCACGGT3’	32
B9	5’ATGGCTCAGC3’	32
B10	5’CAGGCACTAG3’	32
