# Supplementary material for: Transcriptomic Profiling during the Post-Harvest of Heat-Treated Dixiland Prunus persica Fruits: Common and Distinct Response to Heat and Cold
Source: PLoS One. 2012 Dec 6;7(12):e51052. doi: 10.1371/journal.pone.0051052 (PMC3516522; doi:10.1371/journal.pone.0051052)
Supplement: Table S6 — Primers employed for qRT-PCR validation. (PDF) [file pone.0051052.s006.pdf]

Table S6. Primers employed for qRT-PCR validation and analysis under cold storage.

Primer	Forward	Reverse
I3	5’CCCCGCACTCGTACTCTAAC3’	5’GGGTACTCGAACACAACATCC3’
I4	5’GCCTTCCAGGTCCGTTATG3’	5’TGGCAGCAGAAACCCTAGAC3’
I10	5’ATGCAGCCTCAACCAGAGAG3’	5’CGAACTACGTGGGAAAATGG3’
I11	5’TGTCGAGAGACAGTAGCCACA3’	5’GTCCCTGGTGAGAACTTGAATG3’
I12	5’TCAGGGTCCAGACCATTCTC3’	5’ACGGGTCACCAGTTGAAGTC3’
I16	5’CTGGGGCTATCTACTCCAACTG3’	5’GTCCAAACCAAACAAGCTAAGG3’
I18	5’AGATTGAGGGCCAACTATGAAG3’	5’CTGCTTCAGTGTATAGGCTTGG3’
I23	5’GTGCAAGGAGCAGTTGATGTAG3’	5’TGTACCTCTTCACCCATGTGAC3’
I42	5’GTCACATGAGGAGGCACAGA3’	5’ACCCTCCTACCGCTATTCGT3’
I46	5’CGGACGGAACTTTGTCATCT3’	5’TGTCTTCTTTGGGGACCTTG3’
I51	5’ACAGCAGAGATCAAGAGCAAGG3’	5’CTTAAGCCACACAAACCCAGTC3’
I60	5’TCTCACTCTGCGGTGTCTTG3’	5’CATCATCCCTGTTGTTGGTG3’
R2	5 TTCGATGCGATTGAAGACTG’3’	5’CTTCCTGTAATGCTTCTTGG3’
R7	5’CAGAGCAAAGGAATCAAGTG3’	5’ATAGCCCATCGTATCTCTGA3’
R8	5’CTACAAGGGGTTCATTCAAAGC3’	5’GTCCTGCCCTTCATTTCTATTG3’
R12	5’GGAACTGATCCTCGTTCTGG3’	5’GGGCATGAGTGACACAAGGT3’
R14	5’CATATGGCAGCATGTAGTTCGT3’	5’CTTCCTCATCCTTCCTCCTTTT3’
R20	5’CGGTGCACTAGTAGTTGGACAT3’	5’GTTACTTGGGAAGCACTTGGAC3’
R36	5’GGCCTCTACCGGATTTCAA3	5’ACCTCTCCAGCGTCTGTATCTT3’
R42	5’ACTGCTGGTTCTGATGGTTCTT3’	5’TAGTTCTTCGCGGTAGTTGGAT3’
R44	5’CCTCCTCACATTCTCTGCTTCTC3	5’CAGAGGCTGAGGGCTTTGTAGTA3’
O1	5’ATGCAGGGTGGCATTGTAG3’	5’TGGGATGGAAGATGAACAGC3’
O3	5’CCACCATGCTCAATTCTCTC3’	5’GATCCTGTTCCTTGGTGGTT3’
O9	5’GGGAAGTACAAGAGTGTGGAGCAC AGAG’3	5’CTTCTCCTCTGCTTCTTTCTCCACCAG TG’3
